# Prognostic Genes of Breast Cancer Identified by Gene Co-expression Network Analysis

**DOI:** 10.3389/fonc.2018.00374

**Published:** 2018-09-11

**Authors:** Jianing Tang, Deguang Kong, Qiuxia Cui, Kun Wang, Dan Zhang, Yan Gong, Gaosong Wu

**Affiliations:** ^1^Department of Thyroid and Breast Surgery, Zhongnan Hospital of Wuhan University, Wuhan, China; ^2^Department of General Surgery, Zhongnan Hospital of Wuhan University, Wuhan, China; ^3^Department of Thyroid and Breast Surgery, Tongji Hospital, Huazhong University of Science and Technology, Wuhan, China; ^4^Department of Biological Repositories, Zhongnan Hospital of Wuhan University, Wuhan, China

**Keywords:** breast cancer, weighted gene co-expression network analysis (WGCNA), prognosis, GEO, TCGA

## Abstract

Breast cancer is one of the most common malignancies. The molecular mechanisms of its pathogenesis are still to be investigated. The aim of this study was to identify the potential genes associated with the progression of breast cancer. Weighted gene co-expression network analysis (WGCNA) was used to construct free-scale gene co-expression networks to explore the associations between gene sets and clinical features, and to identify candidate biomarkers. The gene expression profiles of GSE1561 were selected from the Gene Expression Omnibus (GEO) database. RNA-seq data and clinical information of breast cancer from TCGA were used for validation. A total of 18 modules were identified via the average linkage hierarchical clustering. In the significant module (*R*^2^ = 0.48), 42 network hub genes were identified. Based on the Cancer Genome Atlas (TCGA) data, 5 hub genes (CCNB2, FBXO5, KIF4A, MCM10, and TPX2) were correlated with poor prognosis. Receiver operating characteristic (ROC) curve validated that the mRNA levels of these 5 genes exhibited excellent diagnostic efficiency for normal and tumor tissues. In addition, the protein levels of these 5 genes were also significantly higher in tumor tissues compared with normal tissues. Among them, CCNB2, KIF4A, and TPX2 were further upregulated in advanced tumor stage. In conclusion, 5 candidate biomarkers were identified for further basic and clinical research on breast cancer with co-expression network analysis.

## Introduction

Breast cancer is the most frequently diagnosed malignancy and the second leading cause of cancer death in females worldwide, accounting for 30% of cancer diagnoses and 14% of cancer death. In 2017, it was estimated that nearly 252,710 new cases were diagnosed in the United States, with ~40,610 deaths (1). Therapeutic strategies of breast cancer have been markedly improved. A number of treatments such as surgery, chemotherapy, radiotherapy, hormone therapy, and targeted therapy are available for breast cancer (2). However, the patients with distant metastases were usually diagnosed with a late stage and nearly incurable (3). Moreover, 30% patients diagnosed with early stage were easy to recur in distant organs even after surgery of removing the primary tumor (4). The classification of breast cancer affects treatment decision and prognosis: hormone-based therapy for ER+ patients; targeted therapy for HER2+ patients; and poorly differentiated cancer often has the worse prognosis (5–7).

Inheritance plays an important role in the development of breast cancer. BRCA1 and BRCA2 are 2 biomarkers which are currently used clinically to assess the familial breast cancer risk. BRCA-associated breast cancer has relatively distinct pathologic characteristics. Up to 20% women with triple-negative breast cancer present BRCA mutations, while BRCA mutations occur less common in general population (8, 9). HER2 expression was found to be upregulated in over 30% patients with breast cancer (10). Previous data suggested that high HER2 levels not only indicated prognostic value, but also affected treatment decisions. Lapatinib and trastuzumab presented dramatically therapeutic effects in patients with HER2-positive breast cancer (11, 12). Expression levels of hormone receptors (ER/PR) predicted the efficacy of endocrine therapies, and their upregulation was often associated with a favorable prognosis (13). Ki-67 was reported to be associated with disease-free survival (14). High CXCR4 levels were associated with lymph node metastasis and distant metastasis (15). Despite the substantial improvements in the treatment of breast cancer, to date, the ability to treat the advanced ones is still limited due to the lack of precise molecular targets for breast cancer (16). Therefore, it is important to explore the molecule mechanisms involved in the occurrence and development of breast cancer. More novel candidate genes are needed to improve the early diagnosis and treatment decisions.

Co-expression analysis is a powerful technique to construct free-scale gene co-expression networks. The weighted gene co-expression network analysis (WGCNA) was widely used to analyze large-scale data sets and to find modules of highly correlated genes. WGCNA was successfully used to explore the associations between gene sets and clinical features, and to identify candidate biomarkers (17). Thus, we described the correlation patterns among genes through a systematic biology method based on WGCNA and identified novel biomarkers associated with breast cancer prognosis.

## Materials and methods

### Data procession

**Figure 1:**
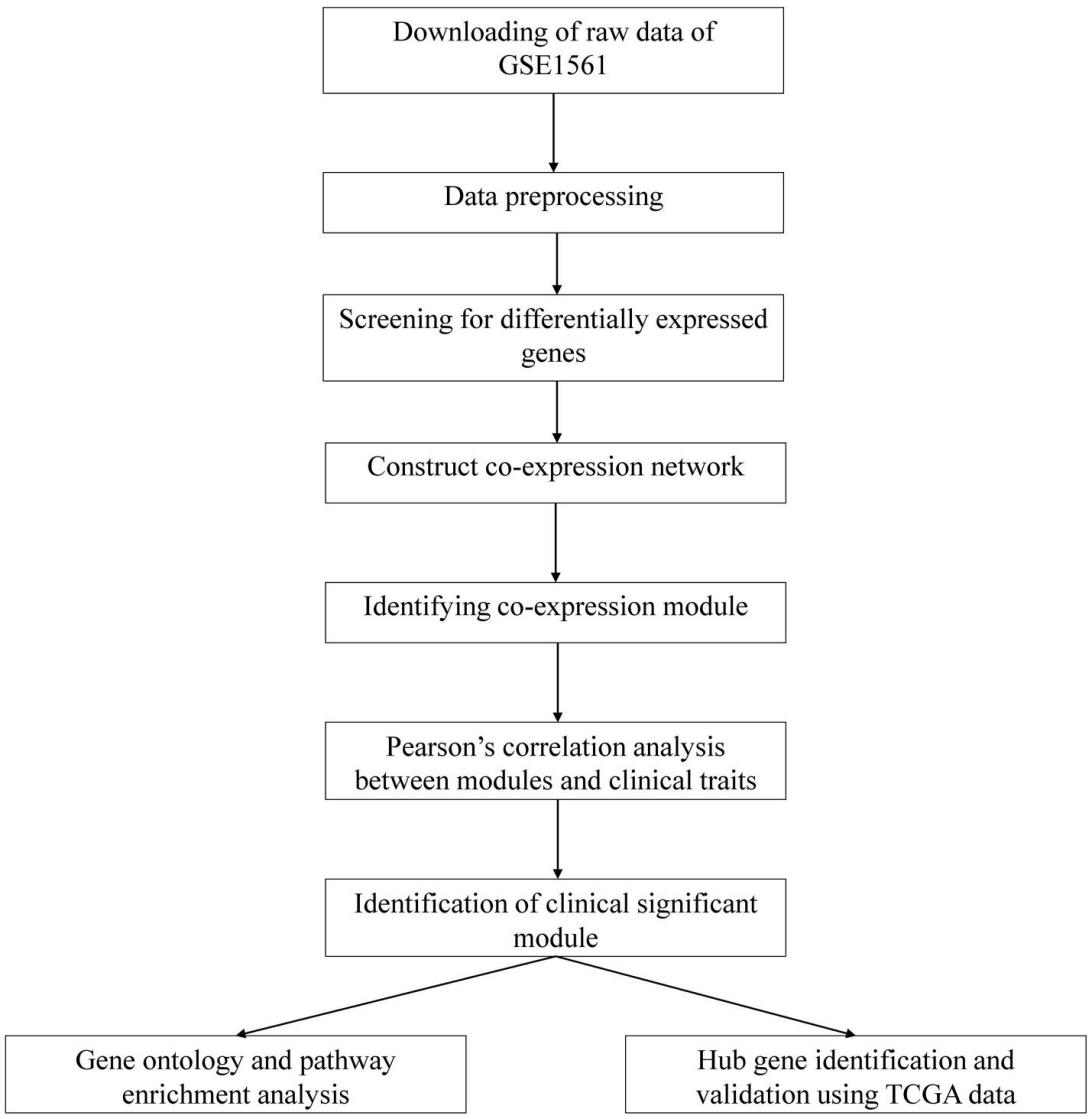
Flow chart of data preparation, processing, analysis, and validation.

A workflow of this study was indicated in Figure 1. The gene expression profiles of GSE1561 (https://www.ncbi.nlm.nih.gov/geo/query/acc.cgi?acc=GSE1561) submitted by Richard Iggo et al. was downloaded from the Gene Expression Omnibus (GEO) database. The GSE1561 was an expression profiling based on GPL96 platform (Affymetrix Human Genome U133A Array) and contained 49 samples. Most patients had 2 trucut biopsies taken, and both biopsies were analyzed from 2 tumors to test the reproducibility of the technique. Repeat amplifications and duplicate biopsies clustered together suggested that biological variation was greater than technical variation in this data set. The results of immunohistochemistry (IHC) also suggested the high quality of this data set (18). Robust Multi-array Average (RMA) algorithm in affy package within Bioconductor (http://www.bioconductor.org) in R was used to preprocess the gene expression profile data. After background correction, quantile normalization and probe summarization, the data set with 12,413 genes was further processed, and the top 50% most variant genes by analysis of variance (6,206 genes) were selected for WGCNA analysis.

### Co-expression network construction

After validation, the expression data profile of these 6,206 genes were constructed to a gene co-expression network using WGCNA package in R (Supplementary Data Sheet 1) (17). The analysis was performed as described previously (17).

The adjacency matrix a_*ij*_ which calculated the connection strength between each pair of nodes was calculated as follows:

$${\text{s}}_{ij}=\;|\text{cor}({\text{x}}_{i},{\text{x}}_{j})|{\text{a}}_{ij}={{\text{S}}_{ij}}^{\beta }$$

Where X_*i*_ and X_*j*_ were vectors of expression value for gene i and j, s_*ij*_ represented the Pearson's correlation coefficient of gene i and gene j, a_*ij*_ encoded the network connection strength between gene i and gene j. In the presented study, the power of β = 9 (scale free *R*^2^ = 0.95) was selected as the soft-thresholding parameter to ensure a scale-free network. In the co-expression network, genes with high absolute correlations were clustered into the same module. WGCNA method not only considers the association between the 2 connected genes, but also takes associated genes into account. Modules were also identified via hierarchical clustering of the weighting coefficient matrix. To further identify functional modules in the co-expression network with these 6,206 genes, the topological overlap measure (TOM) representing the overlap in shared neighbors, was calculated using the adjacency matrix.

$$TOM_{i,j}=\frac{\sum_{K=1}^{N}A_{i,k}\;\cdot \;A_{k,j}+{\text{A}}_{i,j}}{\text{min }\left(K_{i},K_{j}\right)+1-A_{i,j}}$$

Where *A* is the weighted adjacency matrix given by $A_{ij}=\;|cor(x_{i,}x_{j}){|}^{\beta }$ and β= 9 is the soft thresholding power. According to the TOM-based dissimilarity measure with a minimum size (gene group) of 30 for the gene dendrogram, average linkage hierarchical clustering was conducted, and genes with similar expression profiles were classified into the same gene modules using the DynamicTreeCut algorithm.

### Identification of clinical significant modules

Two approaches were used to identify modules associated with clinical information of breast cancer. First, module eigengenes (MEs) were defined as the first principal component of each gene module and the expression of MEs was considered as a representative of all genes in a given module. The correlation between MEs and clinical trait was calculated to identify the clinical significant module. In addition, the gene significance (GS) was defined as mediated *p*-value of each gene (GS = lgP) in the linear regression between gene expression and the clinical traits. Then, the module significance (MS) were defined as the average GS of all the genes involved in the module.MS was measured to incorporate clinical information into the co-expression network. Module significance (MS) was defined as the average absolute gene significance measured for all genes in a given module.

### Gene ontology and pathway enrichment analysis

DAVID (http://david.abcc.ncifcrf.gov/) is a database for annotation, visualization and integrated discovery. Gene Ontology (GO) and KEGG pathway analysis of differentially expressed mRNAs were carried out using DAVID (version 6.8) online tools: functional annotation. The ontology contains three categories: biological process (BP), molecular function (MF), and cellular component (CC). Enriched GO terms and KEGG pathways were identified according to the cut-off criterion of adjusted *P* < 0.001.

### Hub gene identification and validation

The connectivity of genes was measured by absolute value of the Pearson's correlation. Genes with high within-module connectivity were considered as hub genes of the modules (cor.geneModuleMembership > 0.8). Hub genes inside a given module tended to have a strong correlation with certain clinical trait, which was measured by absolute value of the Pearson's correlation (cor.geneTraitSignificance > 0.2). To validate the hub genes, the clinical information and RNA sequencing data of breast cancer were obtained from the Cancer Genome Atlas Project database (TCGA, https://cancergenome.nih.gov/). The mRNA sequencing data was normalized using edgeR package in R language. The Human Protein Atlas (http://www.proteinatlas.org) was also used to validate the immunohistochemistry of candidate hub genes. The direct link to these images in the human protein atlas are as follows: http://www.proteinatlas.org/ENSG00000112029-FBXO5/tissue/breast#img (FBXO5 in normal tissue); http://www.proteinatlas.org/ENSG00000112029-FBXO5/pathology/tissue/breast$+$cancer#img (FBXO5 in tumor tissue); http://www.proteinatlas.org/ENSG00000157456-CCNB2/tissue/breast#img (CCNB2 in normal tissue); http://www.proteinatlas.org/ENSG00000157456-CCNB2/pathology/tissue/breast$+$cancer#img (CCNB2 in tumor tissue); http://www.proteinatlas.org/ENSG00000090889-KIF4A/tissue/breast#img (CCNB2 in normal tissue); http://www.proteinatlas.org/ENSG00000090889-KIF4A/pathology/tissue/breast$+$cancer#img (CCNB2 in tumor tissue); http://www.proteinatlas.org/ENSG00000065328-MCM10/tissue/breast#img (MCM10 in normal tissue); http://www.proteinatlas.org/ENSG00000065328-MCM10/pathology/tissue/breast$+$cancer#img (MCM10 in tumor tissue); http://www.proteinatlas.org/ENSG00000088325-TPX2/tissue/breast#img (TPX2 in normal tissue); http://www.proteinatlas.org/ENSG00000088325-TPX2/pathology/tissue/breast$+$cancer#img (TPX2 in tumor tissue). Survival analysis of hub genes were performed using Kaplan Meier-plotter (www.kmplot.com) (19).

## Results

### Weighted co-expression network construction and key modules identification

**Figure 2:**
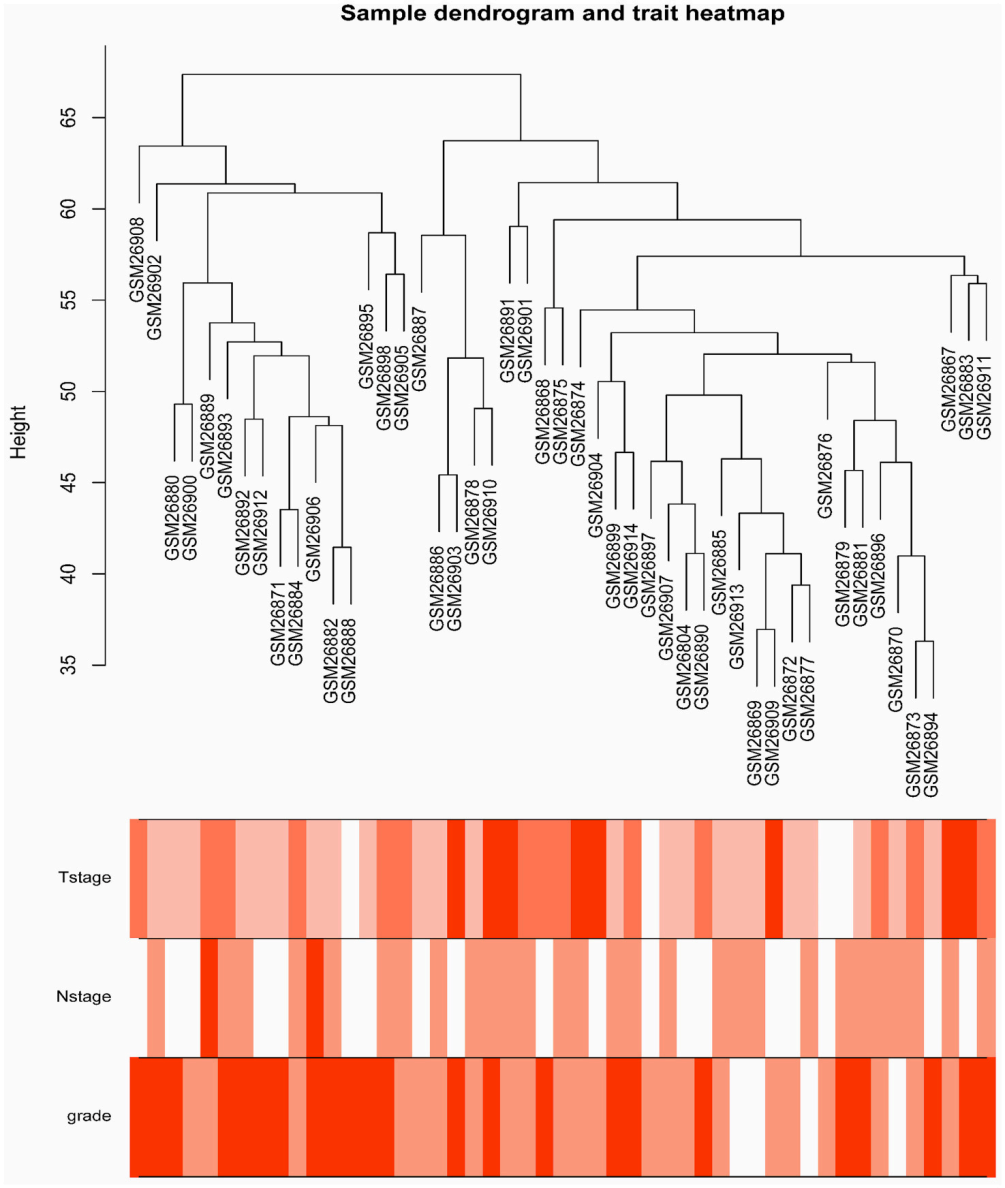
Clustering dendrogram of 49 samples.

**Figure 3:**
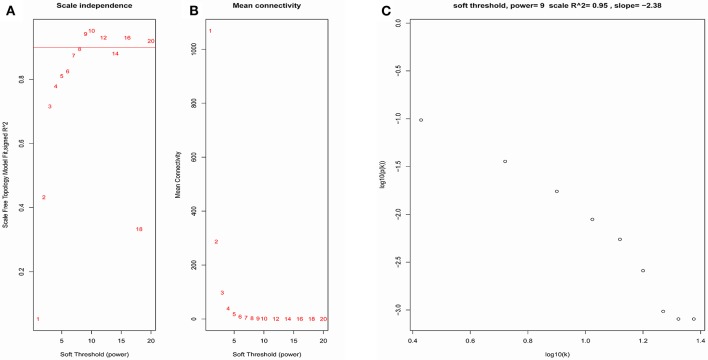
Determination of soft-thresholding power in the WGCNA. **(A)** Analysis of the scale-free fit index for various soft-thresholding powers (β). **(B)** Analysis of the mean connectivity for various soft-thresholding powers. **(C)** Checking the scale free topology when β = 9.

**Figure 4:**
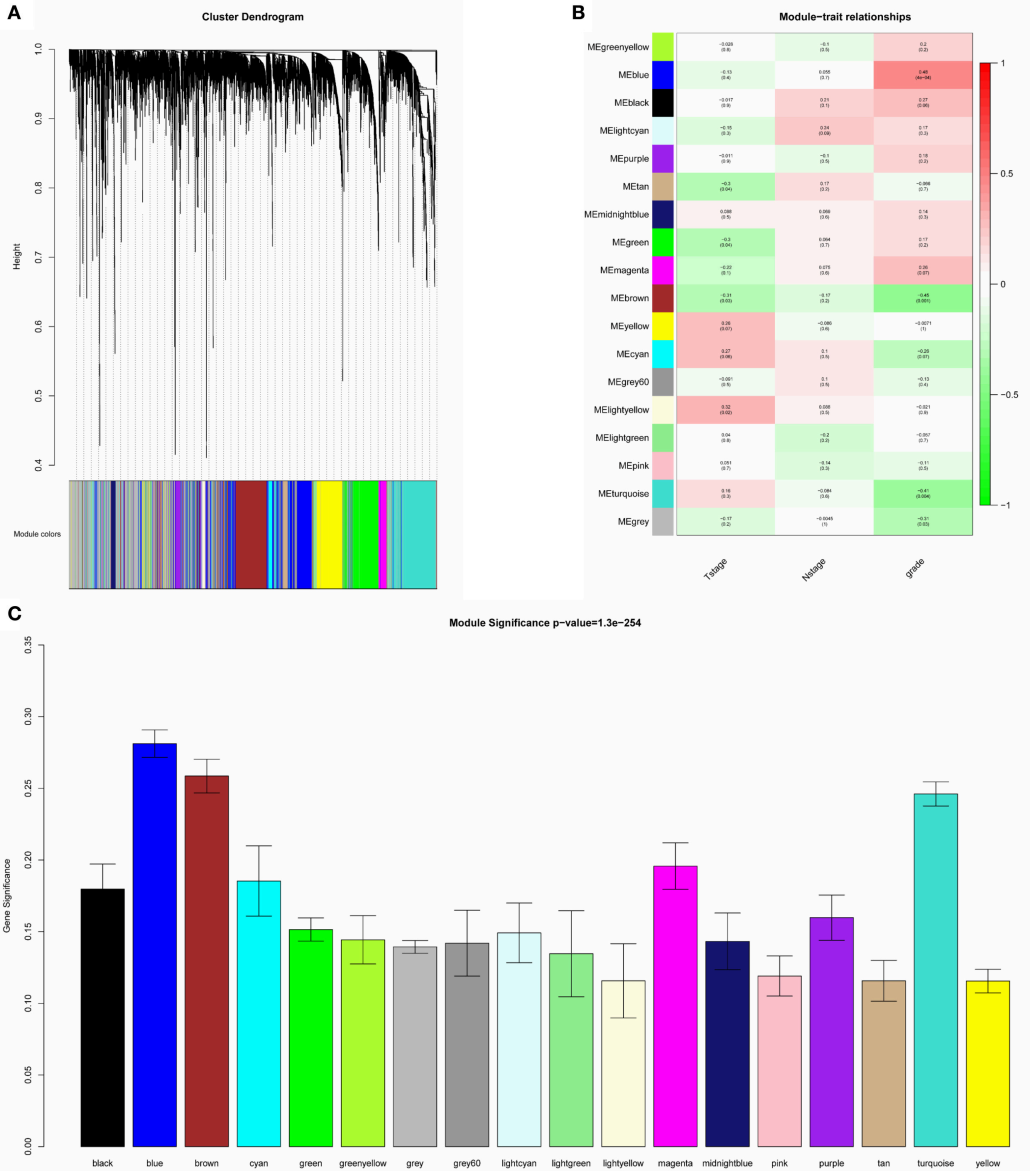
Identification of modules associated with the clinical traits of breast cancer. **(A)** Dendrogram of all differentially expressed genes clustered based on a dissimilarity measure (1-TOM). **(B)** Heatmap of the correlation between module eigengenes and clinical traits of breast cancer. **(C)** Distribution of average gene significance and errors in the modules associated with tumor grades of breast cancer.

The samples of GSE1561 were clustered using average linkage method and Pearson's correlation method (Figure 2). The co-expression analysis was carried out to construct the co-expression network. In this study, the power of β = 9 (scale free *R*^2^ = 0.95) was selected as the soft-thresholding parameter to ensure a scale-free network (Figure 3). A total of 18 modules were identified via the average linkage hierarchical clustering. Blue module was found to have the highest association with tumor grade (Figure 4), and this module was selected as the clinical significant module for further analysis.

### Gene ontology and pathway enrichment analysis

**Figure 5:**
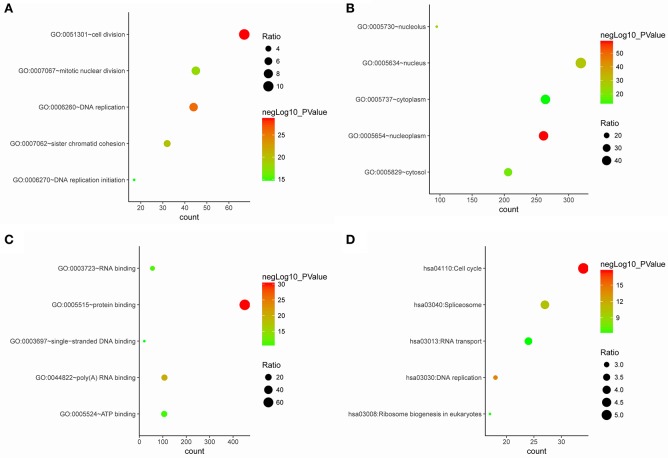
Gene ontology and pathway enrichment analysis of blue module genes. **(A)** Biological process analysis. **(B)** Cellular component analysis. **(C)** Molecular function analysis. **(D)** KEGG pathway analysis.

The genes in the clinical significant module were categorized into 3 functional groups (BP, CC, and MF). Clinical significant module genes in the BP group were mainly enriched in cell division, DNA replication, sister chromatid cohesion, mitotic nuclear division, and DNA replication initiation; The genes in the MF group were mainly enriched in protein binding, poly(A) RNA binding, RNA binding, and ATP binding; the genes in the CC group were significantly enriched in nucleoplasm, nucleus, nucleolus, cytosol, and cytoplasm (Figure 5). According to Kyoto Encyclopedia of Genes and Genomes (KEGG) pathway analysis, our results demonstrated that these genes were mainly involved in cell cycle, DNA replication, spliceosome, ribosome biogenesis in eukaryotes and RNA transport. These results indicated that the clinical significant module genes were mainly involved in mitotic cell cycle process.

### Identification and validation of hub genes

**Figure 6:**
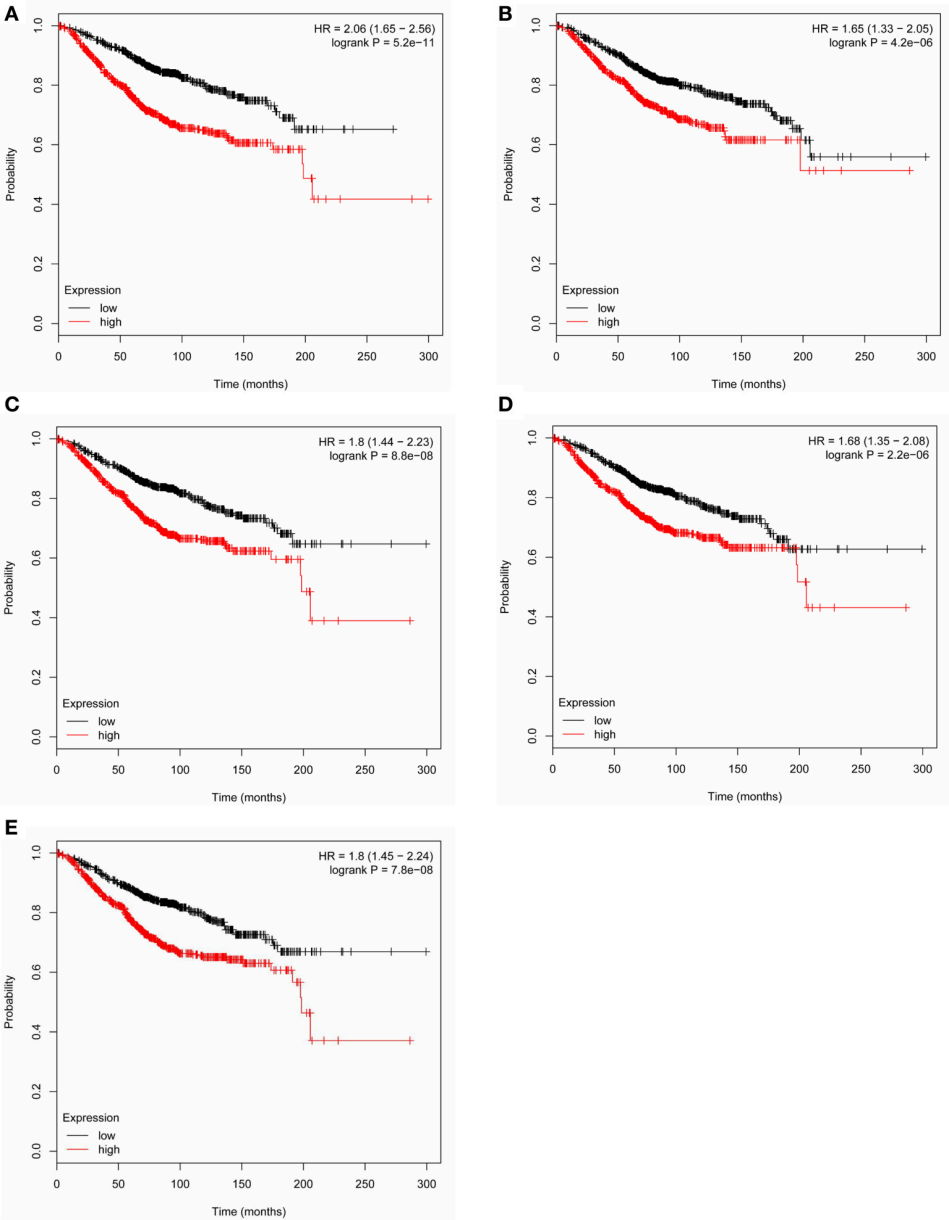
Overall survival of the five hub genes in breast cancer based on Kaplan Meier-plotter. The patients were stratified into high-level group and low-level group according to median expression. **(A)** CCNB2. **(B)** FBXO5. **(C)** KIF4A. **(D)** MCM10. **(E)** TPX2.

**Figure 7:**
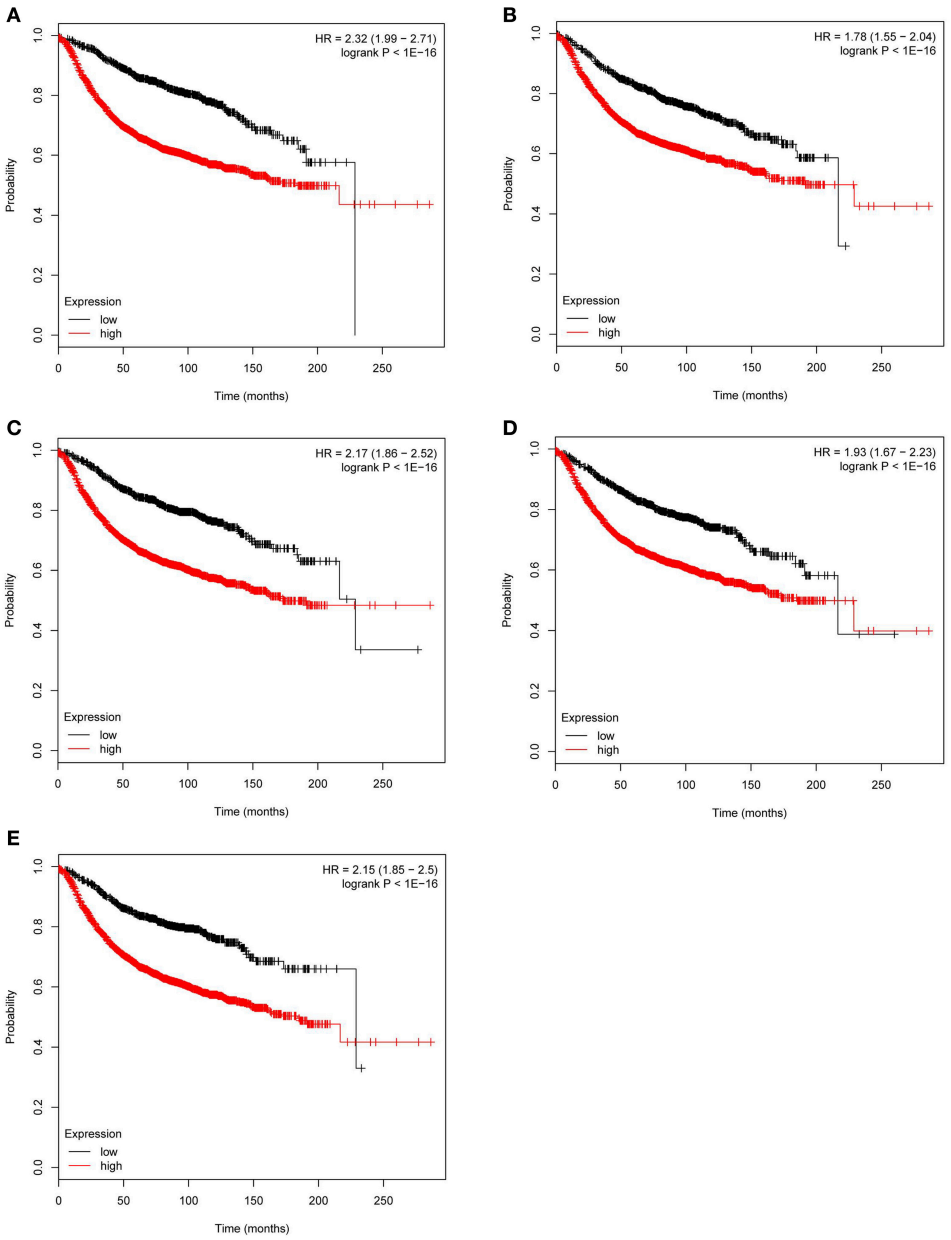
Relapse free survival analysis of the five hub genes in breast cancer based on Kaplan Meier-plotter. The patients were stratified into high-level group and low-level group according to median expression **(A)** CCNB2. **(B)** FBXO5. **(C)** KIF4A. **(D)** MCM10. **(E)** TPX2.

**Figure 8:**
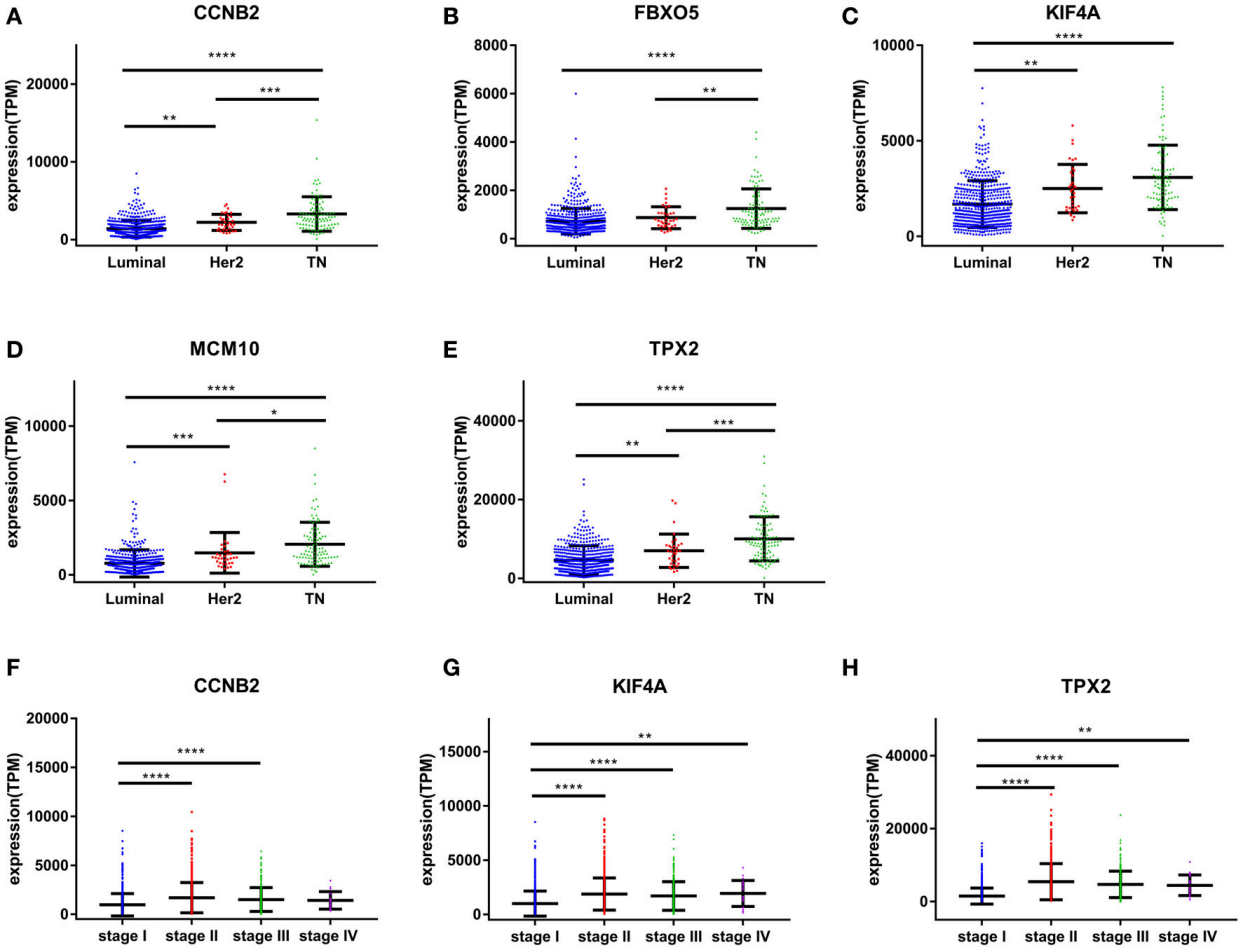
Validation of CCNB2, FBXO5, KIF4A, MCM10, and TPX2. **(A)** The correlation of CCNB2 **(A)**, FBXO5 **(B)**, KIF4A **(C)**, MCM10 **(D)**, and TPX2 **(E)** expression with breast cancer molecular subtypes. **(F)** The correlation of CCNB2 expression with pathological stage. **(G)** The correlation of KIF4A expression with pathological stage. **(H)** The correlation of TPX2 expression with pathological stage.^*^P < 0.05; ^**^P < 0.01; ^***^P < 0.001; ^****^P < 0.0001. One-way analysis of variance (ANOVA) was used to evaluate the statistical significance of differences.

**Figure 9:**
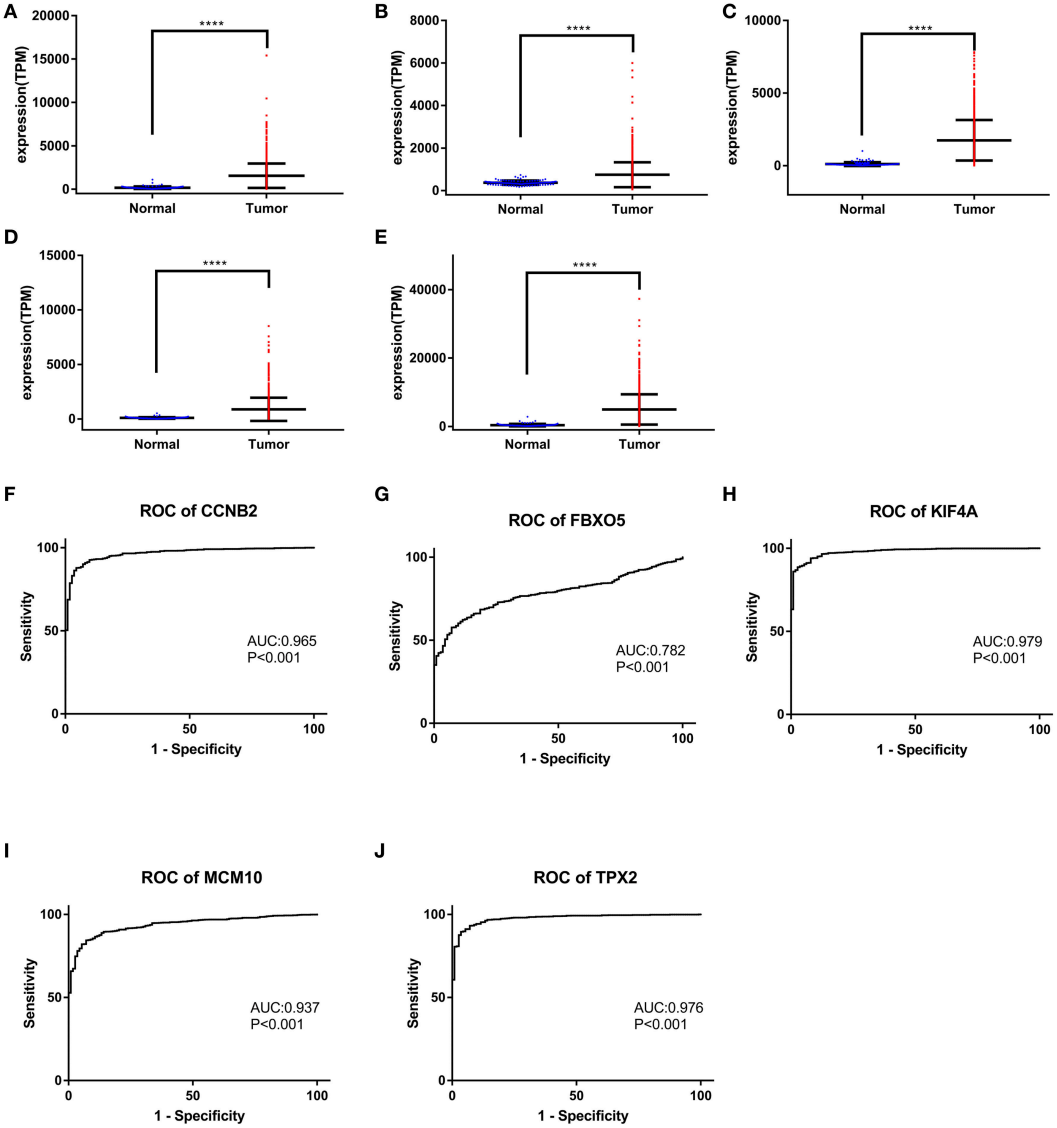
Gene expression levels of CCNB2, FBXO5, KIF4A, MCM10, and TPX2 between normal breast and tumor samples. The mRNA levels of CCNB2 **(A)**, CCNB2 **(B)**, FBXO5 **(C)**, KIF4A **(D)**, and TPX2 **(E)**. ROC curve of CCNB2 **(F)**, FBXO5 **(G)**, KIF4A **(H)**, MCM10 **(I)**, and TPX2 **(J)**. **(A–E)** ^*^P < 0.05; ^**^P < 0.01; ^***^P < 0.001; ^****^P < 0.0001. Two-tailed Student's *t*-tests was used to evaluate the statistical significance of differences.

**Figure 10:**
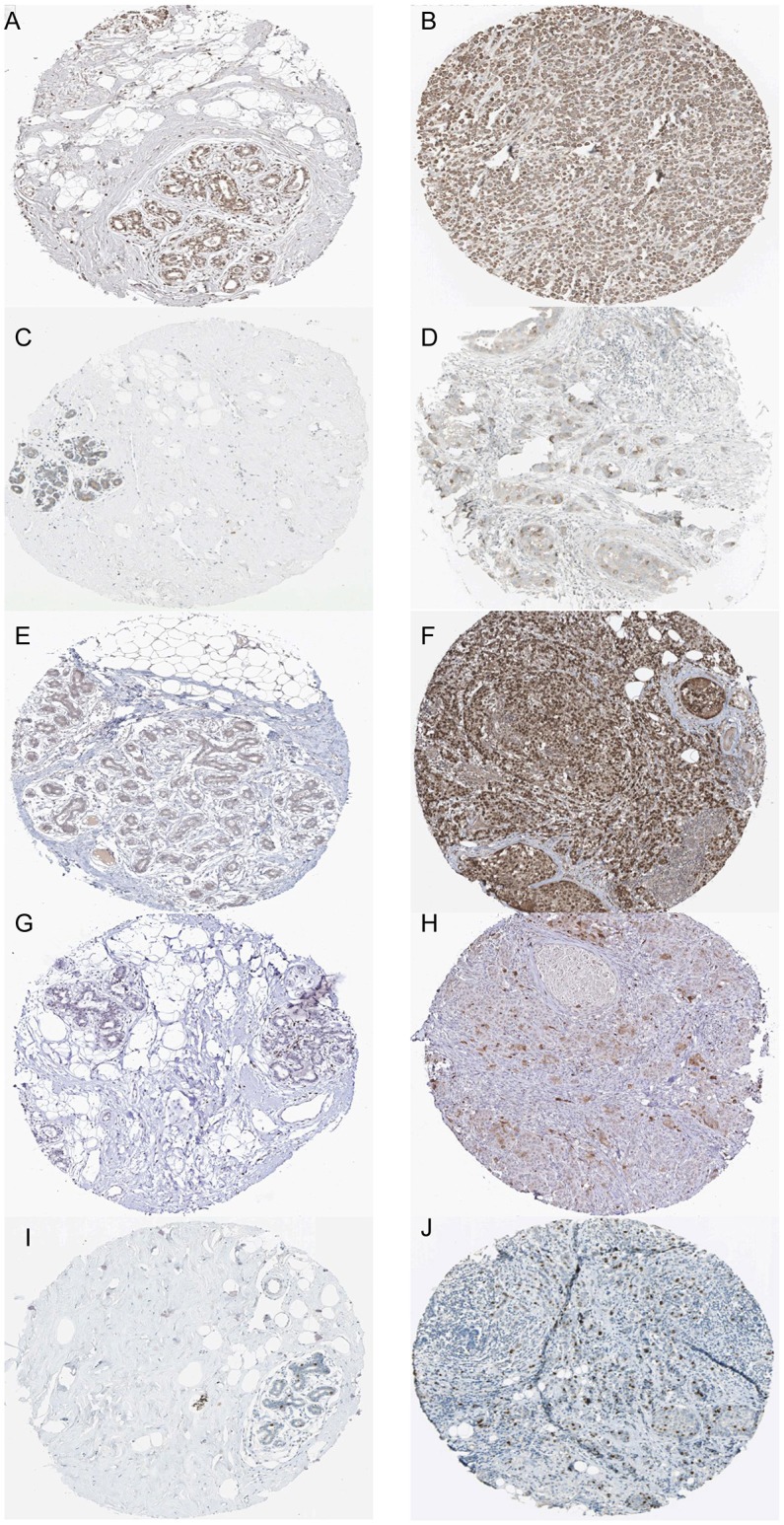
Immunohistochemistry of the five hub genes based on the Human Protein Atlas. **(A)** Protein levels of FBXO5 in normal tissue (staining: medium; intensity: moderate; quantity: >75%). **(B)** Protein levels of FBXO5 in tumor tissue (staining: high; intensity: strong; quantity: >75%). **(C)** Protein levels of CCNB2 in normal tissue (staining: low; intensity: moderate; quantity: <25%). **(D)** Protein levels of CCNB2 in tumor tissue (staining: medium; intensity: strong; quantity: <25%). **(E)** Protein levels of KIF4A in normal tissue (staining: low; intensity: weak; quantity: 25–75%). **(F)** Protein levels of KIF4A in tumor tissue (staining: high; intensity: strong; quantity: >75%). **(G)** Proteins level of MCM10 in normal tissue (staining: not detected; intensity: weak; quantity: <25%). **(H)** Protein levels of MCM10 in tumor tissue (staining: low; intensity: moderate; quantity: <25%). **(I)** Protein levels of TPX2 in normal tissue (staining: medium; intensity: strong; quantity: <25%). **(J)** Protein levels of TPX2 in tumor tissue (staining: medium; intensity: strong; quantity: <25%).

Based the cut-off criteria (|MM| > 0.8 and |GS| > 0.2), 42 genes with high connectivity in the clinical significant module were identified as hub genes. Among them, CCNB2, FBXO5, KIF4A, MCM10, and TPX2 were negatively associated with the overall survival and relapse free survival (Figures 6, 7). Moreover, based on the TCGA data, the expression levels of these 5 genes were significantly higher in tumor tissues, especially in the triple negative breast cancers. The expression of CCNB2, KIF4A, and TPX2 were upregulated in the advanced tumor stages. ROC curve indicated that CCNB2, FBXO5, KIF4A, MCM10, and TPX2 exhibited excellent diagnostic efficiency for normal and tumor tissues (Figures 8, 9). In addition, the protein levels of these 5 genes were significantly higher in tumor tissues compared with normal tissues based on the Human Protein Atlas database (Figure 10). Since these 5 genes were all hub genes in the clinical significant module, they might have a tendency to co-express. Our results of correlation analysis demonstrated a strong correlation of mRNA expression levels between KIF4A and TPX2 (Supplementary Data Sheet 2).

## Discussion

Breast cancer seriously endangers female health, and it is easy to recur even after combined therapy. Although the treatment of breast cancer was improved during the last decades, the ability to treat the advanced ones is still limited due to the lack of precise molecular targets for breast cancer. Therefore, it is important to explore the molecule mechanisms involved in the occurrence and development of breast cancer. Better biomarkers for cancer specific prognosis and progression are highly demanded. In the presented study, we used gene expression datasets from GEO database to screen potential biomarkers related to the progression and prognosis of breast cancer. We also obtained the clinical information and RNA sequencing data of breast cancer from TCGA database for validation.

WGCNA was performed to explore gene co-expression modules associated with progression of breast cancer. A total of 6,206 most variant genes were used to construct co-expression network and 18 modules were identified. Blue module was found to have the highest association with tumor grades and 42 genes with high connectivity were screened out from the module. Among them, CCNB2, FBXO5, KIF4A, MCM10, and TPX2 were negatively associated with the overall survival (Figure 6).

CCNB2, also known as cyclin B2, is a member of cyclin family. CCNB2 was reported to regulate cell cycle by activating CDC2 kinase in eukaryotes, and inhibition of CCNB2 induced cell cycle arrest. CCNB2 was overexpressed in multiple tumors, including bladder cancer, uterine corpus endometrial carcinoma, prostate cancer, and gastric cancer (20–23). In addition, compared with normal controls, the levels of serum circulating CCNB2 are higher in digestive tract cancer and lung cancer patients, and they are found to be significantly associated with tumor stage and metastasis status (24). In invasive breast carcinoma, cytoplasmic CCNB2 protein levels were significantly correlated with a poor disease specific survival. CCNB2 expression level was reported to be an independent prognostic factor for the disease specific survival of breast cancer (25). Our results indicated that CCNB2 was upregulated in breast cancer tissues compared to normal tissues, and that its expression was significantly associated with molecular subtypes of breast cancer and tumor stages (Figure 8). The underlying mechanisms of CCNB2 on tumor progression need to be further clarified.

F-Box Protein 5 (FBXO5) is a key cell cycle regulatory gene which regulates the progression to S phase and mitosis by inhibiting the anaphase promoting complex (APC). FBXO5 is overexpressed in various solid tumors. In the G0 and early G1 phases, the expression of FBXO5 is low, while in the S phase it is upregulated. In ovarian clear cell carcinoma, FBXO5 accumulation was related to mitotic errors with centrosome overduplication and abnormal spindle formation. These findings demonstrated that it might be involved in human cell cycle disorders and genomic stability to promote tumor growth (26–28). In breast carcinoma tissues, FBXO5 induced proliferation through the PI3K/Akt pathway. Overexpression of FBXO5 was reported to correlate with poor prognosis. In addition, PI3K inhibitor reduced FBXO5 expression (29).

The protein encoded by Kinesin family member 4A (KIF4A) was reported to be involved in the intracellular transport of membranous organelles and chromosome integrity during mitosis. In patients with colorectal cancer, KIF4A was upregulated, and downregulation of KIF4A reduced cell proliferation in colorectal cancer cells (30). In hepatocellular carcinoma (HCC) patients, KIF4A overexpression was associated with poorer overall and disease-free survival. In HCC cells, higher levels of KIF4A dramatically increased cellular clonogenic abilities and proliferation, while KIF4A depletion caused a significant augmentation of apoptosis (31). In breast cancer, high KIF4A levels were associated with poor relapse-free survival of ER-positive patients. In tamoxifen-resistant and sensitive breast cancer cells, KIF4A knockdown significantly impeded cellular proliferation and induced apoptosis (32).

Mini-chromosome maintenance complex component 10 (MCM10) is one of the highly conserved mini-chromosome maintenance proteins. MCM10 is bound to chromatin through the interaction with MCM2-7, and plays crucial roles both in initiation and elongation during eukaryotic genome replication (33). For urothelial carcinoma, high MCM10 levels were significantly correlated with advanced tumors stages, vascular invasion, and nodal status. MCM10 overexpression also predicted poor disease-specific survival and inferior metastasis-free survival (34). In our analysis of GSE1561, MCM10 was one of the hub genes in the blue module which was significantly associated with tumor grade (Figure 3). In the validation dataset of TCGA, our results indicated that MCM10 was significantly upregulated in breast tumor tissues, and even higher in the triple negative breast cancer (Figures 8, 9).

Targeting protein for Xenopus kinesin-like protein 2 (TPX2) plays a critical role in chromosome segregation machinery during mitosis (35). It was reported to be overexpressed in multiple tumors: lung cancer, kidney renal clear cell carcinoma, hepatocellular Carcinoma, prostate cancer, and breast cancer (36). TPX2 activates PI3K/Akt pathway and upregulates matrix metalloproteinases (MMP) family members in colon cancer. Previous studies showed that TPX2 expression promoted proliferation, migration, and invasion of liver cancer and breast cancer cells via upregulating expressions of MMP2 and MMP9 (37, 38). In patients with HCC, overexpression TPX2 was correlated with worse prognosis. In addition, knockdown TPX2 in HCC cells strongly reduced cellular proliferation, induced apoptosis and inhibited EMT (39).

Co-expression analysis is a powerful technique for multigene analysis of large-scale data sets. In cancer research, co-expression analyses revealed the mRNA and microRNA expression network in multiple cancers. In the present study, we used WGCNA to construct a gene co-expression network, to measure the relationships between genes and modules, and to explore the relationships between modules and clinical traits. We also screened out a clinical significant module which was associated with the progression of breast cancer. KEGG pathway analysis demonstrated that this module was mostly involved in cell cycle. In addition, 5 hub genes, CCNB2, FBXO5, KIF4A, MCM10, and TPX2 were identified and validated to be associated with the progression and worse prognosis of breast cancer. Our results provided valuable indication for basic and clinical research on breast cancer. The underlying concept of gene co-expression analysis is guilt-by-association. The groups of genes known as co-expression modules were found to maintain a consistent expression relationship independent of phenotype, and might share a common biological role. Similar to the limitations of most other data mining methods, our results of WGCNA can be biased or invalid when dealing with technical artifacts or tissue contaminations (6). To increase the credibility of WGCNA results, TCGA RNA-seq data and IHC data from the Human Protein Atlas database were used for validation. While due to the limitation of the database, the related IHC of each sample can't be found, tumor and normal samples were from different patients.

## Author contributions

JT, YG, and GW reviewed relevant literature and drafted the manuscript. DK, KW, DZ, and QC conducted all statistical analyses. All authors read and approved the final manuscript.

### Conflict of interest statement

The authors declare that the research was conducted in the absence of any commercial or financial relationships that could be construed as a potential conflict of interest.
