# Supplementary material for: Prognostic Genes of Breast Cancer Identified by Gene Co-expression Network Analysis
Source: Front Oncol. 2018 Sep 11;8:374. doi: 10.3389/fonc.2018.00374 (PMC6141856; doi:10.3389/fonc.2018.00374)
Supplement: Supplementary file 1 [file Data_Sheet_1.PDF]

```
setwd("")
# Load the WGCNA package
library(WGCNA);
# The following setting is important, do not omit.
options(stringsAsFactors = FALSE);
breast<-read.table("breast.cancer.gene.exprs.txt",header=T,sep="\t",row.names=1)

m.vars=apply(breast,1,var)
breast=breast[which(m.vars>quantile(m.vars, probs = seq(0, 1, 0.25))[2]),]
breast = as.data.frame(breast);
datExpr0 =as.data.frame(t(breast));
gsg = goodSamplesGenes(datExpr0, verbose = 3);
gsg$allOK
sampleTree = hclust(dist(datExpr0), method = "average");
sizeGrWindow(12,9)
#pdf(file = "Plots/sampleClustering.pdf", width = 12, height = 9);
par(cex = 0.6);
par(mar = c(0,5,2,0))
plot(sampleTree, main = "Sample clustering to detect outliers", sub="", xlab="", cex.lab = 1.5,
     cex.axis = 1.5, cex.main = 2)
traitData = read.table("clinic.txt",header=T,sep="\t",row.names=1)
datExpr = datExpr0
datTraits = traitData
sampleTree2 = hclust(dist(datExpr), method = "average")
traitColors = numbers2colors(datTraits, signed = FALSE);
plotDendroAndColors(sampleTree2, traitColors,
                    groupLabels = names(datTraits),
                    main = "Sample dendrogram and trait heatmap")

# Choose a set of soft-thresholding powers
powers = c(c(1:10), seq(from = 12, to=20, by=2))
# Call the network topology analysis function
sft = pickSoftThreshold(datExpr, powerVector = powers, verbose = 5)
# Plot the results:
sizeGrWindow(9, 5)
par(mfrow = c(1,2));
cex1 = 0.9;
# Scale-free topology fit index as a function of the soft-thresholding power
plot(sft$fitIndices[,1], -sign(sft$fitIndices[,3])*sft$fitIndices[,2],
     xlab="Soft Threshold (power)",ylab="Scale Free Topology Model Fit,signed R^2",type="n",
     main = paste("Scale independence"));
text(sft$fitIndices[,1], -sign(sft$fitIndices[,3])*sft$fitIndices[,2],
```

```
        labels=powers,cex=cex1,col="red");
# this line corresponds to using an R^2 cut-off of h
abline(h=0.90,col="red")
# Mean connectivity as a function of the soft-thresholding power
plot(sft$fitIndices[,1], sft$fitIndices[,5],
        xlab="Soft Threshold (power)",ylab="Mean Connectivity", type="n",
        main = paste("Mean connectivity"))
text(sft$fitIndices[,1], sft$fitIndices[,5], labels=powers, cex=cex1,col="red")

# We use the following power for the power adjacency function
beta1=9
Connectivity=softConnectivity(datExpr,power=beta1)-1

par(mfrow=c(1,1))
scaleFreePlot(Connectivity, main=paste("soft threshold, power=",beta1), truncated=F);
net = blockwiseModules(datExpr, power = 9,
                                TOMType = "unsigned", minModuleSize = 30,
                                reassignThreshold = 0, mergeCutHeight = 0.25,
                                numericLabels = TRUE, pamRespectsDendro = FALSE,
                                saveTOMs = TRUE,
                                saveTOMFileBase = "TOM",
                                verbose = 3)

sizeGrWindow(12, 9)
# Convert labels to colors for plotting
mergedColors = labels2colors(net$colors)
# Plot the dendrogram and the module colors underneath
plotDendroAndColors(net$dendrograms[[1]], mergedColors[net$blockGenes[[1]]],
                            "Module colors",
                            dendroLabels = FALSE, hang = 0.03,
                            addGuide = TRUE, guideHang = 0.05)
moduleLabels = net$colors
moduleColors = labels2colors(net$colors)
MEs = net$MEs;
geneTree = net$dendrograms[[1]];

# Define numbers of genes and samples
nGenes = ncol(datExpr);
nSamples = nrow(datExpr);
# Recalculate MEs with color labels
MEs0 = moduleEigengenes(datExpr, moduleColors)$eigengenes
MEs = orderMEs(MEs0)
moduleTraitCor = cor(MEs, datTraits, use = "p");
moduleTraitPvalue = corPvalueStudent(moduleTraitCor, nSamples);
```

```
sizeGrWindow(10,6)
# Will display correlations and their p-values
textMatrix =    paste(signif(moduleTraitCor, 2), "\n(",
                                  signif(moduleTraitPvalue, 1), ")", sep = "");
dim(textMatrix) = dim(moduleTraitCor)
par(mar = c(7, 8.5, 3, 3));
# Display the correlation values within a heatmap plot
labeledHeatmap(Matrix = moduleTraitCor,
                        xLabels = names(datTraits),
                        yLabels = names(MEs),
                        ySymbols = names(MEs),
                        colorLabels = FALSE,
                        colors = greenWhiteRed(50),
                        textMatrix = textMatrix,
                        setStdMargins = FALSE,
                        cex.text = 0.5,
                        zlim = c(-1,1),
                        main = paste("Module-trait relationships"))

GS1=as.numeric (cor (datTraits$grade, datExpr, use="p"))
GeneSignificance=abs (GS1)

ModuleSignificance=tapply (GeneSignificance, moduleColors, mean, na.rm=T)

plotModuleSignificance (GeneSignificance, moduleColors,
                                        ylim=c (0,0.5), main="Module Significance")



modTraitCor = cor(MEs, datExpr, use = "p")
modTraitP = corPvalueStudent(modTraitCor, nSamples)
corbrown=modTraitCor[which(row.names(modTraitCor)=='MEbrown'),]
head(corbrown[order(-corbrown)])

grade = as.data.frame(datTraits$grade);
names(grade) = "grade"
# names (colors) of the modules
modNames = substring(names(MEs), 3)

geneModuleMembership = as.data.frame(cor(datExpr, MEs, use = "p"));
MMPvalue          =          as.data.frame(corPvalueStudent(as.matrix(geneModuleMembership),
nSamples));
```

```
names(geneModuleMembership) = paste("MM", modNames, sep="");
names(MMPvalue) = paste("p.MM", modNames, sep="");

geneTraitSignificance = as.data.frame(cor(datExpr, grade, use = "p"));
GSPvalue = as.data.frame(corPvalueStudent(as.matrix(geneTraitSignificance), nSamples));

names(geneTraitSignificance) = paste("GS.", names(grade), sep="");
names(GSPvalue) = paste("p.GS.", names(grade), sep="");

module = "blue"
column = match(module, modNames);
moduleGenes = moduleColors==module;
sizeGrWindow(7, 7);
par(mfrow = c(1,1));
verboseScatterplot(abs(geneModuleMembership[moduleGenes, column]),
                   abs(geneTraitSignificance[moduleGenes, 1]),
                   xlab = paste("Module Membership in", module, "module"),
                   ylab = "Gene significance for grade",
                   main = paste("Module membership vs. gene significance\n"),
                   cex.main = 1.2, cex.lab = 1.2, cex.axis = 1.2, col = module)
# Calculate topological overlap anew: this could be done more efficiently by saving the TOM
# calculated during module detection, but let us do it again here.
dissTOM = 1-TOMsimilarityFromExpr(datExpr, power = 6);
# Transform dissTOM with a power to make moderately strong connections more visible in
the heatmap
plotTOM = dissTOM^6;
# Set diagonal to NA for a nicer plot
diag(plotTOM) = NA;
# Call the plot function
sizeGrWindow(19,19)
TOMplot(plotTOM, geneTree, moduleColors, main = "Network heatmap plot, all genes")

# Recalculate module eigengenes
MEs = moduleEigengenes(datExpr, moduleColors)$eigengenes
# Isolate weight from the clinical traits
grade = as.data.frame(datTraits$grade);
names(grade) = "grade"
# Add the weight to existing module eigengenes
MET = orderMEs(cbind(MEs, grade))
# Plot the relationships among the eigengenes and the trait
sizeGrWindow(5,7.5);
par(cex = 0.9)
par(mar = c(20, 8.5, 3, 3))
```

```
plotEigengeneNetworks(MET, "", marDendro = c(0,4,1,2), marHeatmap = c(3,4,1,2), cex.lab =
0.8, xLabelsAngle
                      = 90)
# Plot the dendrogram
sizeGrWindow(6,6);
par(cex = 1.0)
plotEigengeneNetworks(MET, "Eigengene dendrogram", marDendro = c(0,4,2,0),
                      plotHeatmaps = FALSE)
# Plot the heatmap matrix (note: this plot will overwrite the dendrogram plot)
par(cex = 1.0)
plotEigengeneNetworks(MET, "Eigengene adjacency heatmap", marHeatmap = c(3,4,2,2),
                      plotDendrograms = FALSE, xLabelsAngle = 90)

person=cor(datExpr,use = 'p')
corr<-TOM
Colors<-mergedColors
colnames(corr)<-colnames(datExpr)
rownames(corr)<-colnames(datExpr)
names(Colors)<-colnames(datExpr)
colnames(person)<-colnames(datExpr)
rownames(person)<-colnames(datExpr)
umc = unique(mergedColors)
lumc = length(umc)
for (i in c(1:lumc)){
  if(umc[i]== "grey"){
    next
  }
  ME=MEs[, paste("ME",umc[i], sep="")]
  par(mfrow=c(2,1), mar=c(0.3, 5.5, 3, 2))
  plotMat(t(scale(datExpr[,Colors==umc[i]])),nrgcols=30,rlabels=F,rcols=umc[i],
main=umc[i], cex.main=2)
  par(mar=c(5, 4.2, 0, 0.7))
  barplot(ME, col=umc[i], main="", cex.main=2,ylab="eigengene expression",xlab="array
sample")
}

TOM = TOMsimilarityFromExpr(datExpr, power = 9);
module = "brown";
# Select module probes
probes = colnames(datExpr)
inModule = (moduleColors==module);
modProbes = probes[inModule];

# Select the corresponding Topological Overlap
```

```
modTOM = TOM[inModule, inModule];
dimnames(modTOM) = list(modProbes, modProbes)

cyt = exportNetworkToCytoscape(
   modTOM,
   edgeFile = paste("CytoscapeInput-edges-", paste(module, collapse="-"), ".txt", sep=""),
   nodeFile = paste("CytoscapeInput-nodes-", paste(module, collapse="-"), ".txt", sep=""),
   weighted = TRUE,
   threshold = 0.02,
   nodeNames = modProbes,
   nodeAttr = moduleColors[inModule]
);



names(datExpr)
names(datExpr)[moduleColors=="blue"]
probes = names(datExpr)

geneInfo0 = data.frame(substanceBXH = probes,
                        moduleColor = moduleColors,
                        geneTraitSignificance,
                        GSPvalue)
# Order modules by their significance for weight
modOrder = order(-abs(cor(MEs, grade, use = "p")));
# Add module membership information in the chosen order
for (mod in 1:ncol(geneModuleMembership))
{
   oldNames = names(geneInfo0)
   geneInfo0 = data.frame(geneInfo0, geneModuleMembership[, modOrder[mod]],
                          MMPvalue[, modOrder[mod]]);
   names(geneInfo0) = c(oldNames, paste("MM.", modNames[modOrder[mod]], sep=""),
                        paste("p.MM.", modNames[modOrder[mod]], sep=""))
}
# Order the genes in the geneInfo variable first by module color, then by geneTraitSignificance
geneOrder = order(geneInfo0$moduleColor, -abs(geneInfo0$GS.grade));
geneInfo = geneInfo0[geneOrder, ]

write.csv(geneInfo, file = "geneInfo.csv")
```
