# Supplementary figures and images for: Prognostic Genes of Breast Cancer Identified by Gene Co-expression Network Analysis
Source: Front Oncol. 2018 Sep 11;8:374. doi: 10.3389/fonc.2018.00374 (PMC6141856; doi:10.3389/fonc.2018.00374)

**Pearson's correlation=0.917**

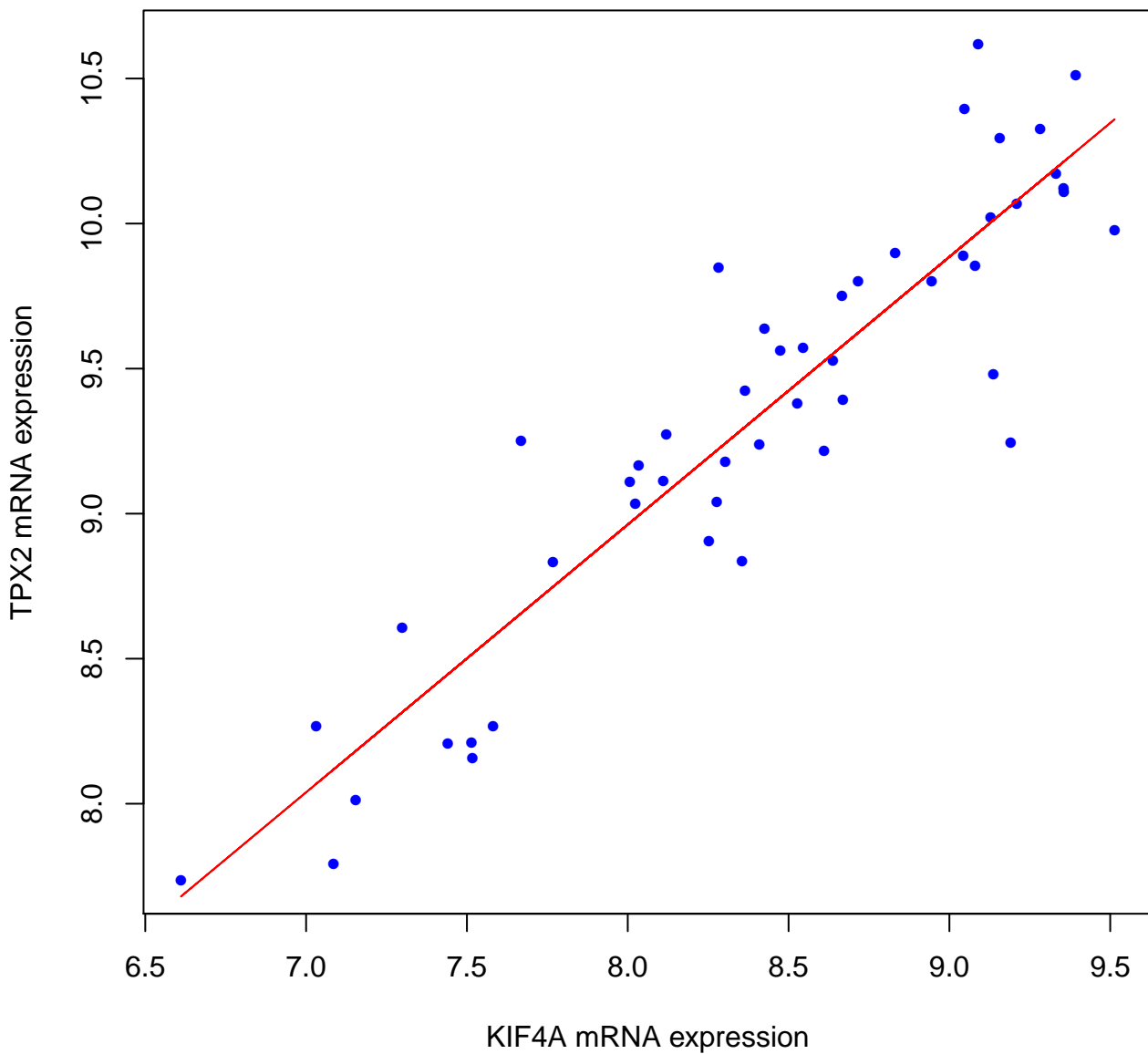

Supplement: Supplementary file 2 [file Data_Sheet_2.PDF]
